# Improvement of alexithymia in patients treated in mental health services for personality disorders: a longitudinal, observational study

**DOI:** 10.3389/fpsyt.2025.1558654

**Published:** 2025-03-26

**Authors:** Hanna Sayar, Theresa Wilberg, Ingeborg Ulltveit-Moe Eikenæs, Andreas Ekberg, Kai Leitemo, Katharina Teresa Enehaug Morken, Eileen Oftedal, Siri Omvik, Dag Anders Ulvestad, Geir Pedersen, Elfrida Hartveit Kvarstein

**Affiliations:** ^1^ Department of Psychology, Faculty of Social Sciences, University of Oslo, Oslo, Norway; ^2^ Institute for Clinical Medicine, Faculty of Medicine, University of Oslo, Oslo, Norway; ^3^ Department of Research and Innovation, Division of Mental Health and Addiction, Oslo University Hospital, Oslo, Norway; ^4^ Department of Addiction Treatment, Division Mental Health and Addiction, Oslo University Hospital, Oslo, Norway; ^5^ Nydalen Mental Health Center, Division of Mental Health and Addiction, Oslo University Hospital, Oslo, Norway; ^6^ Section for Group Therapy, Lovisenberg Diaconal Hospital, Oslo, Norway; ^7^ Department of Addiction Medicine, Haukeland University Hospital, Bergen, Norway; ^8^ Department of Clinical Psychology, University of Bergen, Bergen, Norway; ^9^ Group Outpatient Clinic, Stavanger District Psychiatric Center, Division of Adult Mental Health Care, Stavanger University Hospital, Stavanger, Norway; ^10^ Department of Caring and Ethics, Faculty of Health Services, University of Stavanger, Stavanger, Norway; ^11^ Department of Welfare and Participation, Western Norway University of Applied Sciences, Bergen, Norway

**Keywords:** alexithymia, personality disorders, treatment, longitudinal, improvement, borderline personality disorder, avoidant personality disorder

## Abstract

**Background:**

The majority of mental health services include patients with personality disorder (PD) and comorbid conditions. Alexithymia, a psychological construct referring to difficulties in identifying and describing internal mental states, may represent a challenge to the psychotherapeutic treatment of patients with PD. This study aimed to investigate the prevalence of alexithymia among patients in specialized PD mental health services, differences according to PD severity and PD type, and the longitudinal course of alexithymia during treatment.

**Methods:**

The study included 1,019 patients treated in specialized PD treatment units, with 70% of them with personality difficulties above the PD diagnostic threshold [borderline PD, 31%; avoidant PD, 39%; PD not otherwise specified (PD-NOS), 15%; other PDs, 15%; and more than one PD, 24%]. Alexithymia was measured repeatedly throughout treatment using the Toronto Alexithymia Scale (TAS-20) self-report questionnaire. Supplementary outcomes included global psychosocial function and health-related life quality. Linear mixed models were applied for data analysis.

**Results:**

Alexithymia was highly prevalent in the sample: 53% of subjects reported high levels and 20% moderate levels. The TAS-20 subscale Difficulty Identifying Feelings was more strongly associated with borderline PD, while the subscale Difficulty Describing Feelings was more closely linked to avoidant PD. For all TAS subscales, poorer abilities were associated with more severe PD, higher levels of anxiety and depression, and poorer psychosocial functioning and life quality. Both alexithymia and measures of psychological functioning improved significantly during treatment with moderate effect sizes regardless of initial PD status. In total, 19% of the patients reported full remission of alexithymia.

**Conclusion:**

Alexithymia is a common problem among patients with PDs and is associated with mental health difficulties and psychosocial dysfunction, with rates varying across PD type and severity. The study demonstrates moderate improvement of alexithymia during treatment in specialized PD mental health services. Further research should evaluate the effectiveness of different treatments and interventions in reducing alexithymia among PD patients.

## Introduction

Experiencing and making use of our emotional experiences is a vital part of human functioning and psychological well-being. For individuals with alexithymia, however, this fundamental ability is impaired. The concept of alexithymia, meaning “having no words for emotions”, was coined by Nemiah and Sifneos (1) to describe the lack of ability to recognize and describe one’s emotions, along with an externally oriented cognitive style with a restricted fantasy life. It is theorized to comprise three aspects: *difficulties identifying feelings* (DIF), which represents reduced awareness of different affect states; *difficulties describing feelings* (DDF), which involves the verbal capacity necessary for emotional social interaction through language; and *externally oriented thinking* (EOT), which refers to the reflective processing of emotional states (2). The 20-item self-report questionnaire Toronto Alexithymia Scale (TAS-20; 3) is one of the most widely used methods to evaluate aspects of alexithymia in clinical practice and research. Although the concept of alexithymia was originally developed in a psychosomatic context, its features are recognizable in more recent concepts applied to personality pathology such as personality functioning, emotional awareness, mentalization, and as an aspect of emotional dysregulation (4–7).

Personality disorders (PDs) are, by definition, characterized by pervasive and inflexible patterns of thought, emotion, behavior, and interpersonal style that hinder social and occupational functioning and cause great distress. In developed countries, the prevalence of PDs in the adult population approximates 10% (8). In clinical samples, frequencies of 40%–92% have been reported (9), with borderline PD (BPD) and avoidant PD (AvPD) being the most dominant (8, 10).

### Personality disorders and alexithymia

Compared with the vast amount of studies that have established a link between alexithymia and mental health disorders (11–13), the number of studies that explore alexithymia in PDs is relatively small. To date, studies indicate that alexithymia is associated with the overall number of PD diagnoses and PD traits (14–16), along with the severity of personality disturbance in terms of impaired personality functioning, primitive object relations and defenses, and insecure attachment (17–19). Among specific PDs, alexithymia has been associated with AvPD across studies (14, 16, 20, 21). Honkalampi et al. (22) found that levels of alexithymia among patients with cluster C PDs persisted independent of remission of comorbid major depressive disorder (MDD). For BPD, however, study results have been somewhat inconsistent. While some studies have associated BPD traits with alexithymia (17, 23, 24), Nicolò et al. (16) and De Rick et al. (25) demonstrated only weak or no such associations. There is still a need for research on how aspects of alexithymia are represented in the common PDs in large and representative clinical samples, and on the relationship between alexithymia and the severity of PD.

### Alexithymia and psychotherapy

Psychotherapy is the recommended treatment for PDs. In typical formats, psychotherapy depends on abilities that essentially pose as core deficits for alexithymic individuals, namely the ability to discern and verbalize internal experiences and emotions. Therefore, alexithymic patients presumably present with a disadvantage in a therapeutic setting. Studies across different patient populations and types of interventions have nonetheless demonstrated that alexithymia is partially modifiable through psychological interventions (26–28). With regard to PDs, however, few studies have been conducted, and the majority are based on BPD. Some of these have demonstrated moderate to large improvements in alexithymia following treatment in patients with BPD (29, 30). In contrast, a pilot study of dialectical behavior therapy (DBT) for 21 forensic inpatients with BPD found no detectable change in alexithymia (31). With regard to AvPD, two pilot studies (N = 22 treatment completers) were conducted with significant improvement of alexithymia during treatment (32, 33). Taken together, these studies provide preliminary indications that alexithymia is susceptible to improvement in patients with PDs, but further studies including larger samples of PDs may expand our understanding of how different aspects of alexithymia change over time.

### Complexity of disorders and comorbid conditions

Comorbidity with other mental health disorders is common in the clinical presentation of the majority of PDs, in particular in more severe PD conditions (34, 35). Comorbid psychiatric disorders also likely affect the presentation of alexithymia in PDs. De Panfilis et al. (15) found that the overall numbers of PD criteria and BPD criteria were associated with the EOT factor of the TAS-20 only at low/moderate levels of global distress. The authors suggested that the association between alexithymia and PDs may be disguised when distress from comorbid symptomatology is high. In a similar vein, Joyce et al. (17) found that when trait anxiety was controlled for, alexithymia did not predict outcome in a mixed PD patient sample who received intensive treatment for 18 weeks. Honkalampi et al. (22) conducted a 6-month follow-up study of general psychiatric outpatients with MDD and MDD with comorbid cluster C PDs. Although both groups displayed similar levels of alexithymia at the first assessment, patients with MDD and comorbid cluster C PDs experienced a slower reduction in alexithymia symptoms compared to those with MDD alone. Taken together, these studies suggest that the prevalence and trajectory of alexithymia not only vary across PD categories but also are influenced by the patient’s current symptom burden and comorbid disorders.

### The present study

The present study focuses on a clinical sample of patients with different types and severities of PD. It is part of a large, longitudinal, multicenter research project (TREATPD) based on clinical data from the quality register of the Norwegian Network for Personality Disorders (the Network)—a cross-regional collaboration of PD treatment units within specialized mental health services (10, 36). It includes a naturalistic sample of patients with personality difficulties all of whom are treated in group therapy-based psychotherapy units within a regular, specialized mental health service level.

The primary aim of the study is to investigate the extent of alexithymia in a heterogeneous sample of patients referred to PD treatment and to explore how such problems change during treatment. Secondary aims are to investigate the heterogeneity of alexithymia (DIF, DDF, and EOT) in relation to the most common PD types (BPD and AvPD), PD severity, depressive and anxiety symptoms, presence of major depression, and total number of comorbid symptom disorders at treatment referral. Supplementary investigation includes the longitudinal course of patient-rated health-related life quality and observer-rated psychosocial functioning and their association with levels and change of TAS-20.

## Materials and methods

### Design

The study is an observational, longitudinal study based on data from the quality register of the Network (36). The study is based on data collected in the period June 2017–June 2021.

### Treatment setting

The data collection period included 15 specialized PD treatment units within outpatient adult specialist mental health and addiction services. The treatments provided across the treatment units in the Network range from long-term psychodynamic group therapy to shorter group therapies, evidence-based manualized treatments, of which mentalization-based treatment (MBT) is the most frequent, and various combinations of group therapy approaches such a combination with individual therapy. The treatment setting and the distribution of treatment approaches in relation to different PDs were elaborated in the first TREATPD study (10). The length of treatment offered varied between treatment units and PD conditions. The overall sample (N = 1,019) had a mean treatment duration of 15 months (SD, 9), with a 12% dropout rate (10).

The therapists involved were engaged in multidisciplinary treatment teams of different health professionals, such as psychiatric nurses, social workers, clinical psychologists, and psychiatrists. The Network regularly provides updated courses and conferences on PD assessment procedures and therapeutic principles for all associated therapists. Further elaboration of therapist qualities is given in a separate publication based on the same quality register (37).

### Battery of evaluation instruments

The battery used in the Network included both patient and clinician reports. Interviews were conducted at baseline, while self-reports were collected every 6 months from the start of treatment and throughout treatment. Separate items designed specifically for the Network inquired about sociodemographic factors from patients, such as age, gender, family circumstances, educational background, prior treatment history, and experiences with self-harm or suicidal incidents. Therapists provided data through diagnostic interviews, as well as detailed reports on the nature, duration, and adherence to current treatment.

### Baseline assessment

#### Mental health disorders

Participating treatment units applied common routines for diagnostic assessments recommended within the Network. Semi-structured interviews were performed by clinicians at the units before starting treatment (baseline) for symptom disorders, the Mini International Neuropsychiatric Interview (MINI; 38) for PDs, and the Structured Clinical Interview for Section II DSM-5 Personality Disorders (SCID-5-PD; 39). Clinicians in the Network received training in diagnostic interviews and principles of the Longitudinal, Expert, All-Data (LEAD) procedure (40, 41). Diagnostic classification was confirmed by a specialist in psychiatry/clinical psychology at each unit (**Table 1**). Diagnostic procedures and training are further elaborated in the first TREATPD publication (10).

**Table 1:** Demographics and psychosocial status before starting treatment.

	Frequency/scores in clinical range %	Mean (SD; min–max)
Demographics		
Age		30 (9; 17–67)
Female gender	78	
Living alone	29	
Years of education after mandatory school (age 6–16)		4.2 (2)
Months > 50% work/study in last 6 months		2.6 (3; 0–12)
EQ-5D-3L problems with activity/work/study	78	
VAS		49 (50; 0–98)
GFS		52 6; 30–75)
Previous treatment experience		
Previous treatment in mental health services	82	
More than two treatment series	57	
First treatment < 18 years of age	59	
Previous hospital admissions	32	
Severity of condition		
Total number of SCID-5-PD criteria		11 (6; 0–39)
No PD	30	
PD-NOS	15	
EQ-5D-3L symptoms of anxiety/depression	99	
GAD-7		13 (4.8; 0–21)
PHQ-9		18 (5.3; 1–27)
Total number of symptom disorders		1.2 (1.3; 0–8)
Specific PD categories		
Schizoid and schizotypal	1	
Paranoid	9	
Antisocial	2	
Narcissistic and histrionic	2	
Borderline	31	
Avoidant	39	
Dependent	6	
Obsessive-compulsive	6	
Specific symptom disorders		
Mood disorders	75	
Current major depression	51	
Anxiety disorders	47	
PTSD	13	
Substance use disorder	10	
Eating disorder	8	
ADHD	7	
OCD	4	
Other disorders	3	

Demographic status of patients at referral for treatment in the period 2017–2021. Clinical ranges for EQ-5D-3L items are indicated by % with scores > 1.

VAS, visual analog scale; GFS, Global Functioning Scale; SCID-5-PD, Structured Clinical Interview for section II DSM-5 Personality Disorders; PD, personality disorder; PD-NOS, PD not otherwise specified; GAD-7, Generalized Anxiety Disorder-7; PHQ-9, Patient Health Questionnaire; PTSD, post-traumatic stress disorder; ADHD, attention-deficit hyperactivity disorder; OCD, obsessive-compulsive disorder.

#### Anxiety and depressive symptom load

The Generalized Anxiety Disorder-7 (GAD-7) is a patient self-report of anxiety symptoms with seven items rated on a 0–3 scale (42). Sum scores ≥10 indicate a possible anxiety disorder (43). The Patient Health Questionnaire, Depression (PHQ-9) is a patient self-report on depression symptoms with nine items rated on a 0–3 scale (44, 45). Sum scores ≥10 indicate clinically relevant depressive symptoms (46, 47). In this study, we report baseline symptom levels.

### Repeated assessments

#### Primary outcome measures

The Toronto Alexithymia scale (TAS-20; 3) is a 20‐item questionnaire rated on a 1–5 scale (completely disagree to completely agree). It comprises three domains inquiring about aspects of the alexithymia construct: emotional awareness, expression, and reflective capacity; 1) DIF (e.g., item 1: *I am often confused about what emotion I am feeling*), 2) DDF (e.g., item 2: *It is difficult for me to find the right words for my feelings*), and 3) EOT [e.g., item 19 (reversed): *I find examination of my feelings useful in solving personal problems*]. A total TAS-20 score is based on all 20 items. Commonly referenced intervals for TAS-20 scores are as follows: low alexithymia, mean total score < 2.6; intermediate alexithymia, mean total score 2.6–3.0; high alexithymia, mean total score > 3.0. A psychometric investigation of a Norwegian version of the TAS-20 revealed acceptable psychometric properties, although poorer results for EOT (48). In this study, we report the mean total score when investigating the longitudinal course of alexithymia, but due to the different psychometric qualities, we chose to perform predictive analyses based on the three domains.

The Difficulties in Emotion Regulation Scale (DERS; 5, 49, 50) consists of 36 items organized in six subscales, each item rated on a 1–5 scale (almost never to almost always). In order to validate the information derived from the TAS-20, aspects of alexithymia were also assessed using the DERS subscale 1, Lack of Emotional Clarity, which has five items, e.g., item 5: *I have difficulty making sense of my feelings.* Mean scores ≥2 indicated the presence of problems. Lack of emotional clarity and the DIF aspect of the TAS-20 shared 41% of the variance (r = 0.64) in this sample (Pedersen et al.)^1^. This study included DERS assessments at baseline and the last 6 months of treatment/last registered assessment.

#### Supplementary global outcome measures

Two supplementary measures were included to demonstrate the global impact of alexithymia and included both patient and clinician evaluations. The patient self-report, Health-related Life quality (EQ-5D-3L; 51) has five items rated on a 3-point scale (no problems to extreme problems) and a visual analog scale (VAS), ranging in health state from worst to best possible (scores 0–100). Descriptive baseline data included the two EQ-5D-3L items: problems with activity/study/work and symptoms of anxiety/depression (% with score > 1). Mean VAS scores (global burden of disease) in general population studies range from 80 to 89 (52). In this study, VAS > 60 was considered to indicate non-clinical levels. The VAS evaluation was performed at baseline and repeated every 6 months during treatment.

Clinicians rated a modified version of the Global Assessment of Functioning Scale (GAF; 53), termed the Global Functioning Scale (GFS; 54) aided by a specially designed interview guide. In a study by Pedersen et al. (55), the reliability of the original split version of the GAF was found to be acceptable. The GFS gives two scores ranging from 1 to 100, representing symptom severity and social impairment. The lower of the two scores is reported (56). Conventional interpretations of severity are similar to the original GAF with moderate and severe impairment (clinical levels) indicated by GFS < 60. GFS evaluation was performed at baseline and repeated every 6 months during treatment.

### Sample size

The sample originally retrieved from the quality register included all patients admitted and discharged from treatment between 2017 and 2021 (N_total_ = 1,051). The main analyses in the present study included only patients with available assessment of alexithymia (N_longitudinal study sample_ = 1,019). In addition, a change from the start to the last 6-month phase of treatment was presented (TAS-20 _end of treatment_, n = 513; DERS _end of treatment_, n = 481). Predictor analyses included all patients with available assessments of alexithymia, diagnostic evaluation, and self-reported symptom burden (N_predictoranalyses_ = 875).

### Statistics

All analyses were performed using SPSS Statistics for Windows, Version 27 (57).

#### Longitudinal analyses

Linear mixed models (LMMs) were applied (58, 59). The primary aim was addressed by LMM with the TAS-20 total mean score as the dependent variable. The secondary aim (predictor analyses) included three LMMs with each of the TAS-20 subscales as dependent variables (DDF, DIF, and EOT). The supplementary investigation included two separate LMMs with the dependent variables GFS and VAS. In all models, time (months from baseline) was modeled as a continuous variable. According to log-likelihood estimations, the best-fit model for all the dependent variables included linear time, random intercept and slope, and unstructured covariance (critical values for chi-square statistic: *p* < 0.01).

The secondary predictor analyses included baseline assessments of BPD, AvPD, total number of PD criteria, PHQ-9 sum score, GAD-7 sum score, current major depression, and total number of symptom disorders as independent variables. The possible variance associated with age and gender was also investigated. Predictors were investigated in separate models and together in a final model. **Table 2** reports LMM estimates for change trajectories (intercept and slope), predictor-associated deviation, variance components (intercept and slope), and log-likelihood statistics [Akaike information criterion (AIC)]. Explained variance is the % reduction in variance estimates from the model without the predictor (reference value). Strong inferences are indicated by *p* < 0.01 (fixed effects), % explained variance, and improved model fit (AIC). Supplementary LMM investigation with GFS and VAS models included LMM analyses of the TAS-20 total mean score as a time-varying, continuous predictor variable.

**Table 2:** TAS-20 subscales; longitudinal analyses and predictor investigations.

LM models: N = 875	Predictors	Fixed effects: estimates for linear trajectories				Variance components		The goodness of model fit
		Intercept (SE)	*p*	Slope (SE)	*p*	Intercept (ref)/% explained variation	Slope (ref)/% explained variation	AIC
*TAS-20 subscales*								
Identify feelings		3.2 (0.3)	<0.001	−0.02 (0.003)	<0.001	0.65 (ref)*	0.001 (ref)*	4,153
	Male gender	−0.25 (0.08)	<0.001		ns	2%	0%	4,154
	Age	−0.02 (0.003)	<0.001		ns	3%	0%	4,157
	BPD	0.62 (0.07)	<0.001	−0.02 (0.006)	0.01	14%	0%	4,085
	AvPD		ns	0.01 (0.005)	0.06	0%	0%	4,161
	PD crit.	0.05 (0.01)	<0.001		ns	15%	0%	4,067
	GAD-7	0.08 (0.01)	<0.001	−0.002 (0.001)	0.01	25%	0%	3,956
	PHQ-9	0.06 (0.01)	<0.001		ns	14%	0%	4,085
	Age, gender, all PD predictors, PHQ-9 and GAD-7					32%	0%	3,975
Describe feelings		3.4 (0.03)	<0.001	−0.02 (0.003)	<0.001	0.67 (ref)*	0.001 (ref)*	4,164
	Male gender	0.18 (0.08)	0.02		ns	0%	0%	4,169
	Age	−0.02 (0.003)	<0.001		ns	4%	0%	4,158
	BPD		ns		ns	0%	0%	4,175
	AvPD	0.53 (0.07)	<0.001		ns	9%	0%	4,111
	PD criteria	0.03 (0.01)	<0.001		ns	6%	0%	4,149
	GAD-7	0.03 (0.01)	<0.001		ns	3%	0%	4,099
	PHQ-9	0.03 (0.006)	<0.001		ns	3%	0%	4,159
	Age, gender, all PD predictors, PHQ-9 and GAD-7					18%	0%	4,081
Externally oriented thinking		2.5 (0.02)	<0.001	−0.007 (0.002)	<0.001	0.29 (ref)*	0.000 (ref)*	2,656
	Male gender		ns		ns	0%	0%	2,663
	Age		ns	0.001 (0.0002)	0.01	0%	0%	2,664
	BPD		ns		ns	0%	0%	2,666
	AvPD	0.22 (0.04)	<0.001		ns	3%	0%	2,641
	PD criteria	0.02 (0.003)	<0.001		ns	7%	0%	2,643
	GAD-7	0.02 (0.004)	<0.001		ns	3%	0%	2,626
	PHQ-9	0.03 (0.004)	<0.001		ns	7%	0%	2,637
	Age, gender, all PD predictors, PHQ-9 and GAD-7					10%	0%	2,661

Linear mixed (LM) model estimations with baseline levels (intercept estimate), and monthly change rate (slope estimate) for three dependent variables, separate predictor analyses, and variance estimates for final models including all predictors together. Non-significant differences are indicated by ns (*p* > 0.05). Significant estimates of variation are indicated by * (*p* < 0.05). The goodness of model fit is indicated by the Akaike information criterion (AIC), where smaller values are better.

TAS-20, Toronto Alexithymia Scale; PD, personality disorder; BPD, borderline PD; AvPD, avoidant PD; GAD-7, Generalized Anxiety Disorder-7; PHQ-9, Patient Health Questionnaire.

#### Considerations of unbalanced data

Data collection for the quality register was based on regular administrative clinical routines. Missing assessments may thus be due to randomly occurring failures in the administration and delivery systems. As the data collection was limited to the treatment period, shorter treatment durations naturally caused fewer assessments.

For the overall investigation of possible bias from missing longitudinal data, we included longitudinal analyses as the dependent variable TAS-20 sum score and an independent variable counting the number of assessment points (60). In these analyses, a higher number of assessment points was not associated with a deviating change pattern for the TAS-20 sum score (*p* > 0.05). For a more specific investigation of possible bias due to different treatment durations, we report longitudinal analyses with the TAS-20 sum score as the dependent variable and treatment duration as the independent variable.

The main longitudinal study sample (N = 1,019) included 1,676 measurement occasions, the mean number of assessments per individual was 1.9 (SD 1.1, min 1, and max 8), and 52% had two or more measurement occasions. Longitudinal analyses were, in addition, replicated in a smaller sample of patients with a higher number of assessments (selection of patients with two or more assessments, N = 523; mean number of assessments, 2.8; SD 1.0). The baseline status in the main study sample was similar to that in the smaller sample, but the mean treatment duration was longer in the smaller sample. Considerations about the quality of the data collection are further elaborated in the first TREATPD publication (10).

#### Presentation of change

Longitudinal 0–18-month effect sizes were based on LMM-predicted values and computed according to Cohen’s *d* (61; small effect size: *d* = 0.2, medium *d* = 0.5, and large *d* = 0.8); 18 months was an intermediate approximation adjusted for assessment time points (treatment duration ≤ 18 months: 67%). In addition, a score reduction (termed improvement) from the start to the last 6 months of treatment and the percentage that changed from clinical to non-clinical values during treatment (full remission), were reported for the TAS-20, DERS emotional clarity, GFS, and VAS.

#### Other descriptions

Statistical analyses of start–end change in DERS emotional clarity were based on paired-sample t-tests.

## Results

### Baseline status on treatment referral

Sociodemographic data and social and mental health status are shown in **Table 1**. The majority of the subjects were women in a young adult age group (58% < 30 years), 70% had at least one PD diagnosis, and among them, 24% had two or more PD diagnoses. AvPD and BPD were the most frequent specific PDs. The mean number of SCID-PD-5 criteria for patients with PD was 12.9 (SD 5.7), and the mean number of criteria when below the PD threshold was 3.5 (SD 2.4). Of the patients, 32% reported prior admissions to a psychiatric hospital, and 82% stated they had previous outpatient treatment experience, with 59% before the age of 18 years. Reported health-related problem areas were activity/work/study (78%) and symptoms of anxiety/depression (99%).

In this mixed PD sample, the TAS-20 total mean score when referred to treatment was 2.95 (SD 0.7), with 28% classified as low alexithymia, 20% as intermediate alexithymia, and 53% as high alexithymia. The majority reported scores > 3 on the two TAS-20 subscales DIF (65%) and DDF (71%). A smaller proportion reported scores > 3 on the TAS-20 subscale EOT (24%). A total of 87% had scores > 2 on the DERS emotional clarity.

### Overall change during treatment

**Figure 1:**
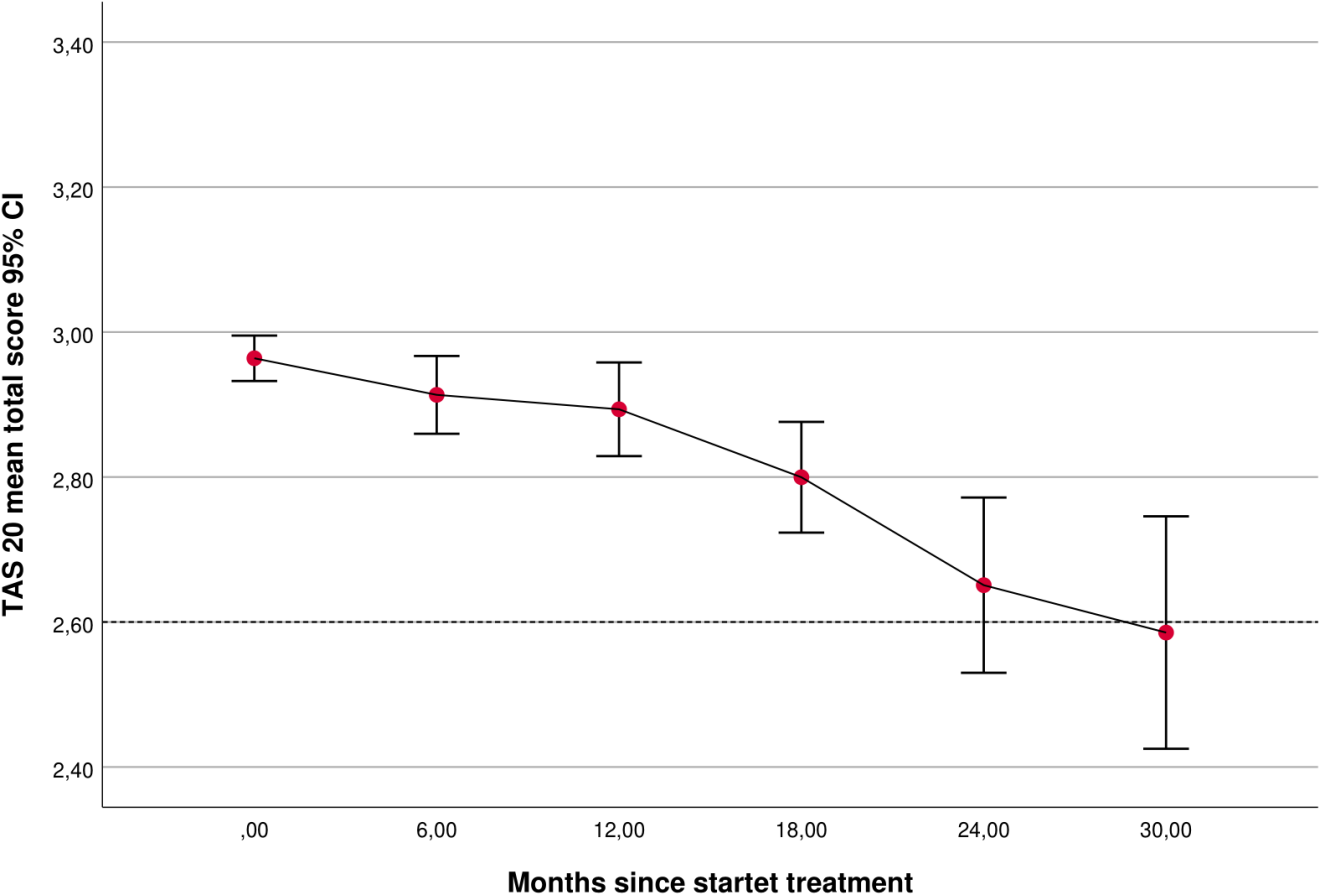
TAS-20 sum scores at different time points during treatment (at referral, time 0: n = 981; 6 months: n = 363; 12 months: n = 265; 18 months: n = 155; 24 months: n = 73, 30 months; n = 30). Within-subject variation is demonstrated by calculated confidence intervals (CIs). TAS-20, Toronto Alexithymia Scale.

In the mixed PD sample, the TAS-20 total mean score improved significantly over time with the LMM change trajectory starting at a mean estimated value of 3.0 (SE 0.03, intercept *p* < 0.001) and decreasing at an estimated rate of 0.01 points per month (SE 0.002, slope *p* < 0.001; **Table 1**; **Figure 1**). Differences in treatment duration (months in treatment) were associated with differences in baseline levels of TAS-20 scores (higher scores associated with longer treatment, *p <* 0.05), but were not associated with significantly deviating rates of longitudinal change in TAS-20 scores (*p* > 0.05).

The longitudinal 0–18-month LMM predicted that the effect size (ES) for the TAS-20 total mean score was moderate (ES_0–18 months,_ 0.5; N = 1,019). The mean score at the last 6-month assessment was 2.7 (SD 0.7). From the start to the last 6 months of treatment, 63% of patients with initially high alexithymia reported TAS-20 improvement and 19% reported full remission of alexithymia (TAS-20 total mean score changed from >3 to <2.6). Similarly, 15% of patients with initial problems of emotional clarity assessed using DERS reported a DERS emotional clarity score < 2, with start–end DERS emotional clarity difference (*p* < 0.001).

### Analyses of TAS-20 subscales and predictors

**Figure 2:**
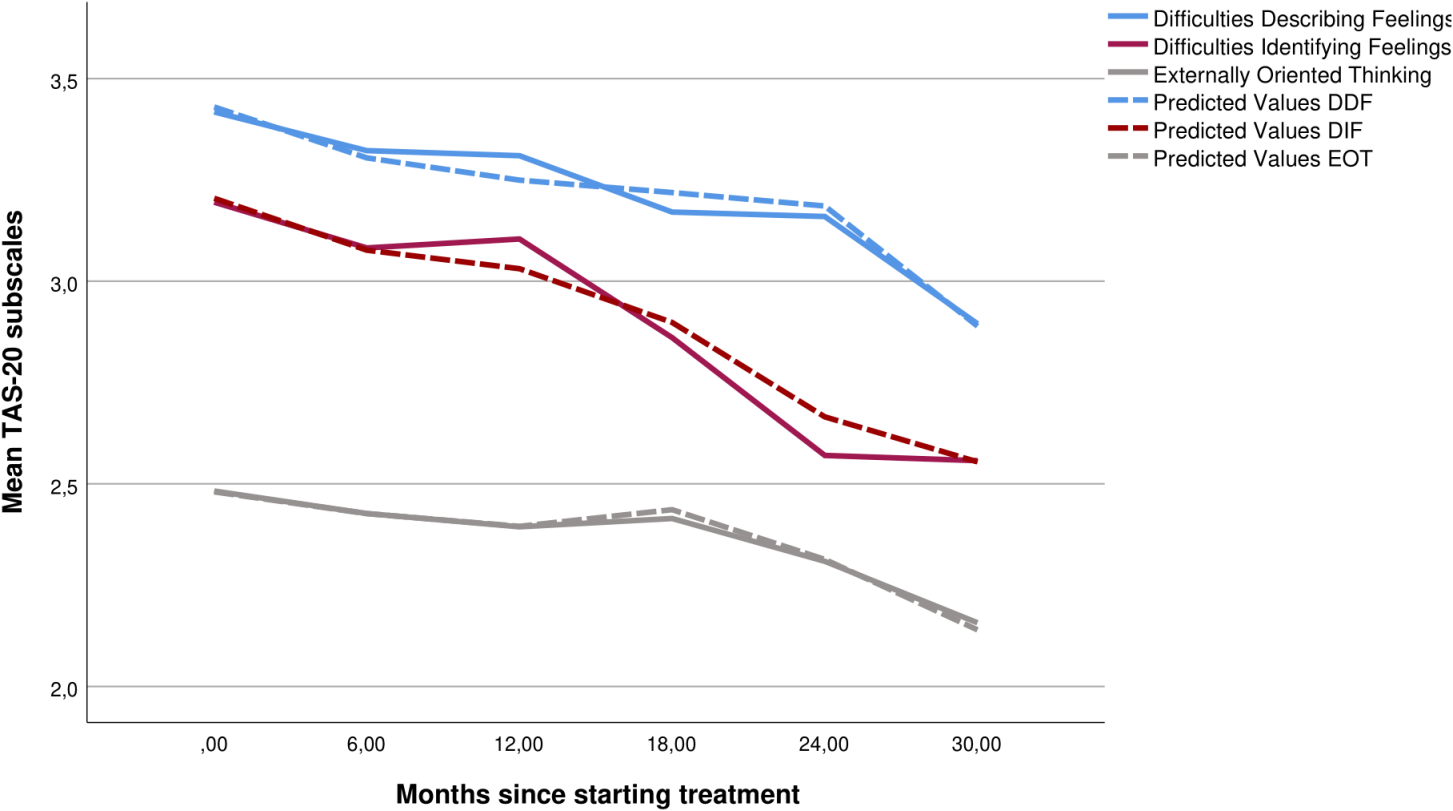
Mean scores (solid line) and LMM predicted values (dashed line) for the three subscales—difficulties describing feelings, difficulties identifying feelings, and externally oriented thinking—at different time points during treatment including trajectories for all patients (N = 875). LMM, linear mixed model.

**Figure 3:**
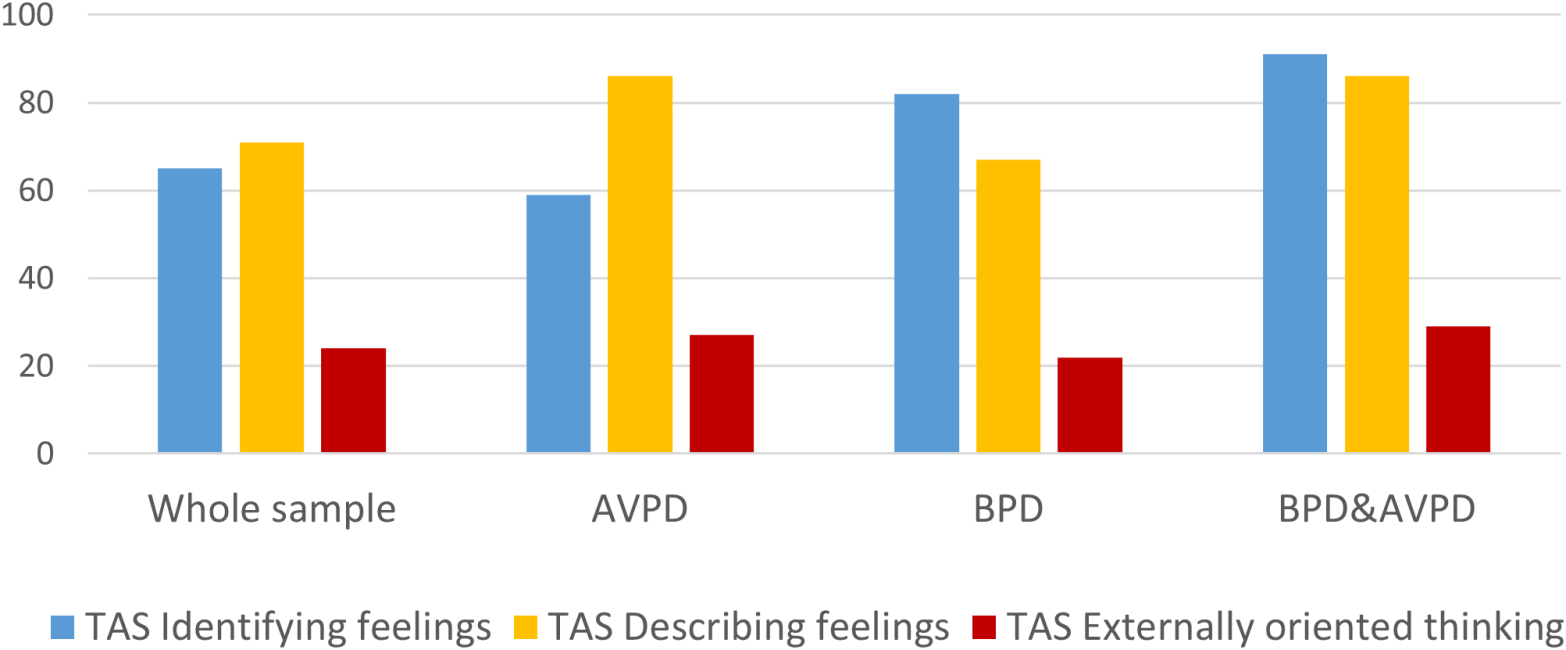
The proportion (%) of patients indicating problems (scores > 2.6) within the three TAS-20 subscale domains at the start of treatment, in the total sample (TAS-20 baseline assessment: N = 985), among patients with avoidant personality disorder (AvPD; n = 266), borderline personality disorder (BPD; n = 199), and AvPD–BPD comorbidity (n = 137). TAS-20, Toronto Alexithymia Scale.

All three TAS-20 subscales improved significantly over time (**Table 2**, **Figure 2**). Intercept and slope variations were significant for all TAS-20 subscales and allowed further investigation of predictors. Overall longitudinal 0–18 months predicted that the effect sizes for the three TAS-20 subscales were small to moderate (ES_0–18 months_: DIF, 0.4; DDF, 0.6; and EOT, 0.4; N = 1,019). **Figure 3** illustrates baseline differences in TAS-20 subscales in patients with AvPD, BPD, and co-occurring AvPD and BPD.

#### TAS-20 subscale: difficulty identifying feelings

In separate models, and maintained in the final model including all predictors, female gender, younger age, BPD, increasing number of PD criteria, and increasing levels of anxiety and depressive symptoms assessed at baseline were all significantly and uniquely associated with poorer ability to identify feelings (**Table 2**). BPD, the number of PD criteria, and symptoms of depression (PHQ-9) explained the largest proportions of intercept variation, while AvPD had no significant unique impact and explained little further intercept variation (**Figure 3**). In the final model, none of the predictors were associated with deviating change over time (*p* > 0.05), and minimal slope variation was explained by these predictors.

#### TAS-20 subscale: difficulties describing feelings

In separate models, and maintained in the final model including all predictors, male gender, younger age, AvPD, increasing number of PD criteria, and increasing levels of anxiety and depressive symptoms were all significantly and uniquely associated with poorer ability to describe feelings (**Table 2**). AvPD explained the largest proportion of intercept variation, while BPD had no significant extra impact and explained little further intercept variation (**Figure 3**). None of the predictors were associated with deviating change over time, and minimal slope variation was explained by this predictor.

#### TAS-20 subscale: externally oriented thinking

In separate models, and maintained in the final model including all predictors, AvPD, increasing number of PD criteria, and increasing levels of anxiety (GAD-7) and depressive symptoms (PHQ-9) were significantly and uniquely associated with more externally oriented thinking (**Table 2**). PD criteria and depressive symptoms explained the largest proportion of intercept variation, while BPD had no significant extra impact and explained little further intercept variation (**Figure 3**). Only age was associated with significantly deviating change over time, indicating that older age was associated with slower improvement.

#### Impacts of major depression and number of symptom disorders

Examination of the two predictors of major depression and the total number of symptom disorders (baseline status) in separate LM models with the three TAS-20 subscales rendered non-significant impacts on intercept levels and change over time (all *p* > 0.05) and no further explanation of improved model fit (AIC). In the final models including these predictors, all other trends remained.

### Global functioning and well-being and associations with the TAS-20

The mean levels of VAS and GFS indicated considerable experienced health burden and impairment upon referral to treatment (**Table 1**). Among patients with high alexithymia at baseline, 91% had GFS scores < 60, and 63% had VAS scores < 60. Significant improvement during treatment was found in the LM models for the two supplementary global outcomes: VAS (LMM estimates: intercept, 49, SE 0.9; slope, 0.5, SE 0.6) and GFS (LMM estimates: intercept, 52, SE 0.3; slope, 0.25, SE 0.3). Among patients with initially high alexithymia, 62% reported VAS improvement and 77% GFS improvement.

Examination of TAS-20 as a time-varying predictor revealed significant interactions with GFS and with VAS. Lower (poorer) levels of GFS were significantly associated with higher (poorer) levels of TAS-20 at all time points. For time = 0 (at referral/baseline), the estimated GFS decrease per one-point TAS-20 increase was −1.3 (SD 0.28, *p* < 0.001). The estimated GFS decrease in monthly change rate per one-point TAS increase was −0.09 (SD 0.03, *p* = 0.003). Correspondingly, at baseline, the estimated VAS decrease per one-point TAS-20 increase was −5.8 (SD 0.8, *p* < 0.001), and the estimated VAS decrease in monthly change rate per one-point TAS increase was −0.26 (SD 0.07, *p* < 0.001).

## Discussion

The current study employed a longitudinal design and aimed to explore alexithymia in a uniquely large, naturalistic clinical sample of patients undergoing treatment from specialized PD mental health services in a Norwegian context. The sample reflects the heterogeneity typical of treatment-seeking samples, representing patients with personality problems of different severity and types. An earlier TREATPD study indicated the high utility of PD treatment within mental health services and an overall improvement in personality functioning across PDs (10). The present study represents a more specific exploration of central emotional capacities, i.e., identification, description, and preoccupation with emotional states, and the relevance of such capacities in a mixed PD sample. The main findings are summarized as follows:

1. Alexithymia was commonly reported: more than half of the sample reported high levels of alexithymia and 20% reported moderate levels of alexithymia. Among the TAS subscales, externally oriented thinking was the least prevalent.
2. For all TAS subscales, poorer abilities were associated with more severe PD and symptoms of anxiety and depression.
3. BPD was particularly associated with difficulties identifying feelings and AvPD with difficulties describing feelings and externally oriented thinking.
4. Alexithymia and problems within all three alexithymia aspects improved during treatment with moderate effect sizes. The change was irrespective of PD condition, other mental disorder comorbidities, initial depressive state, and anxiety symptoms.
5. Global measures indicated considerable health burden and social impairment upon referral to treatment, but significant improvement over time. More severe alexithymia was associated with poorer life quality and psychosocial functioning at all assessment points during treatment.

### Alexithymia—highly prevalent and associated with severity in PD samples

The results align with those of other studies demonstrating moderate to high rates of alexithymia among patients with PDs (14–16, 22). Although the sample in Honkalampi et al. (22) exclusively consisted of patients with cluster C PDs, Nicolò et al. (16) and De Panfilis et al. (15) explored alexithymia in samples with a range of mental health disorders including PDs from general psychiatric outpatient settings. The current sample consisted of individuals from specialized PD treatment units, which further attests to alexithymia being a common difficulty among patients with PDs.

The present study also found that the severity of all alexithymia aspects was closely and uniquely associated with several indicators of condition severity and comorbidity, including the number of PD criteria, symptoms of anxiety and depression, poorer psychosocial functioning, and subjective burden of health issues. Our findings mirror the trends reported in previous studies relating to alexithymia and aspects of PD severity and personality functioning. Joyce et al. (17) found more severe personality disorders, poorer emotional regulation, and more interpersonal problems among 51 psychiatric outpatients with PD and high alexithymia. In a general community sample of 501 individuals, Preece et al. (18) related high alexithymia with poorer emotion regulation abilities. Similarly, in a clinical sample of 56 patients with AvPD, Simonsen et al. (19) found that higher levels of alexithymia were associated with poorer personality functioning. They propose that the level of alexithymia may serve as an indicator of the severity of personality dysfunction in PD populations. These findings have some potentially important clinical implications. They allow clinicians to identify individuals who present with more severe personality pathology and related mental health issues, which allows for more tailored interventions. Furthermore, considering alexithymia as a potential indicator of personality disorder severity may guide clinicians in monitoring and evaluating treatment progress, ensuring a more comprehensive approach to patient care.

### Alexithymia and PD differences

As illustrated in **Figure 3**, the main problems of alexithymia in the current study were represented as identifying and describing feelings, and significant overrepresentation associated with specific PDs was evident. We found that BPD was particularly associated with difficulties in identifying emotions. Similar trends were reported in a study by New et al. (23), who investigated a sample of patients with BPD and AvPD and healthy controls. Studies have shed light on how BPD patients struggle to discern between internal psychological content and external circumstances and to differentiate their own emotions from those of others, which may explain the particular difficulties of this group in identifying their emotional experiences (62–64). AvPD was associated with particular problems with describing feelings—a result also reported in the study of Nicolò et al. (16) based on 388 patients with a range of mental health illnesses and PDs. In an interview-based study focusing on metacognitive functioning among patients with AvPD, Moroni et al. (65) found particular impairment in the mindreading function referred to as “monitoring”. This aspect is theorized to involve the ability to describe internal states and the motivations underlying behaviors and feelings. Millon (66) viewed the difficulties of avoidant patients as stemming from defensive aspects of their personality, which leads to a compartmentalization of emotions through muddling and repression of affect. This may shed light on the incessant fear of criticism and rejection that these patients usually experience when in an emotionally vulnerable position.

We emphasize that, based on the distribution in our current sample, these difficulties cannot be considered PD-specific, and our findings must be interpreted with care. It is noteworthy that in the present study, irrespective of the type of PD, the large majority reported problems from both domains. For example, while more than 80% of patients with AvPD in our sample reported difficulties in the DDF, more than 60% of the BPD patients reported the same. The results indicate that individuals with PDs may struggle with similar difficulties regarding how they relate to and make use of their emotional experiences. At the same time, there are likely nuanced differences in how specific PDs impact and impair different aspects of emotional functioning. The results call for clinicians to tailor therapeutic interventions to the emotional difficulties of each patient.

Externally oriented thinking was less common in our sample and accounted for approximately one-fifth of the sample. Although we found that AvPD was significantly associated with stronger tendencies to externally oriented thinking, differences and numbers were small. We do not know if this result reflects the poorer psychometric properties of this particular construct (48, 67). It is generally recommended that the EOT scale be interpreted with caution.

### Possible effects of comorbidity

Comorbidity has been hypothesized to disguise associations between alexithymia and PDs (15, 22). In the current study, the effect of MDD or the number of symptom disorders on the level of alexithymia or change over time was not significant. However, current symptom distress of anxiety and depression was associated with higher levels of alexithymia, which touches on the ongoing discussion of whether alexithymia and depression represent overlapping constructs (68, 69). The present findings further reinforce the importance of controlling for comorbid symptoms and anxiety when exploring alexithymia in clinical samples.

### Change in alexithymia

A central aim of the current study was to explore improvement in alexithymia during treatment, and the estimated effect sizes indicate an overall trend of moderate change. The majority of patients improved but did not report full remission. Improvement rates were irrespective of initial PD status, symptom severity, and other mental disorder comorbidities. Among those with initial alexithymia, equivalent proportions reported improvement of alexithymia, global functioning, and subjective burden of health during psychotherapy. The current study does not allow us to draw causal conclusions regarding the relationship between improvement in alexithymia and related parameters of psychological well-being. However, the findings suggest that alexithymia may improve over time and that focusing therapeutic interventions on enhancing emotional awareness may improve not only alexithymia but also the overall mental health functioning in individuals with PDs. Further research should investigate potential causal mechanisms between alexithymia and other clinical phenomena.

Previous studies have correspondingly indicated that psychological interventions may improve alexithymia. Cameron et al. (26) found moderate improvements in alexithymia following psychological interventions across mental health disorders more generally. With regard to PD samples, studies indicate that alexithymia may improve from psychological interventions, although the ES varies across studies. In a mixed sample of patients with a range of mental health difficulties, including PDs, who were offered short-term psychodynamic therapy, Ogrodniczuk et al. (27) found a small ES for interpretative therapy and moderate ES for supportive therapy (0.18 and 0.65) in reducing alexithymia. Löf et al. (29) reported small-to-moderate improvements of the three TAS subscales (DIF = 0.52, DDF = 0.29, and EOT = 0.25) in a sample of patients with BPD in an 18-month MBT program. Similarly, McMain et al. (70) explored change in alexithymia in a sample of 80 patients with BPD who received DBT and general psychiatric management (GPM) for 1 year and found small-to-moderate ES for change in the three factors of the TAS-20 (DIF = 0.51, DDF = 0.25, and EOT = 0.19). Rossi et al. (30) reported moderate and large ES (0.63 and 0.89) for change in alexithymia across two groups of patients with BPD who received metacognitive interpersonal therapy (MIT) and structured clinical management (SCM) over 12 months. Regarding AvPD, Wilberg et al. (33) found a large ES (0.84) for the total TAS score in a sample of patients with AvPD in a 2-year individual and group therapy program based on MBT and MIT. Also in a similar program of 12–18-month treatment duration in patients with AvPD, a large ES (0.97) for the total TAS score was found (32).

Differences in ES across studies may be caused by a number of factors, such as differences in study designs, type and length of therapy provided, and the contexts of patient recruitment. In the present study, the change in alexithymia may be underestimated due to the design of the study, which did not include an assessment at the end of treatment but rather in the last 6-month phase of treatment. Nonetheless, the trends across studies indicate that alexithymia is responsive to psychological interventions and that a range of treatments may be helpful in this pursuit. Still, little is known about specific interventions that may benefit alexithymic patients. In their review, Cameron et al. (26) found that interventions specifically targeting alexithymia appeared to yield better results than those focused on general psychological improvement. Although the treatment provided in the current study did not target alexithymia specifically, the majority of patients received some kind of group therapy-based treatments such as MBT that have been developed for PD populations in particular. Group therapy provides patients with the unique possibility of being in a setting where they can observe how others explore and verbalize their emotions. The unpredictable nature of the format encourages participants to engage in novel interpersonal situations where they utilize their emotional experiences. It is likely that alexithymic difficulties to some extent are addressed in this type of treatment, even though it is not an explicit focus. There is a need for more knowledge of how targeted treatments compare to broader therapeutic approaches in alexithymic PD populations and how group therapy compares to individual therapy.

The importance of treating alexithymia in patients with PDs is highlighted by our finding that alexithymia was strongly related to poorer psychosocial functioning and a higher subjective burden of health issues across all stages of treatment. While other studies have emphasized the considerable societal burden of PDs (71, 72), we did not find other studies related to measures of health-related life quality and alexithymia. Although observational studies do not allow us to draw causal inferences about the impacts of alexithymia, they nonetheless shed light on the considerable burden that is associated with such emotional problems and therefore also the potential benefits of addressing these issues during treatment.

## Limitations

The present study had several limitations. Although the diagnostic procedures generally held a high standard, as it was completed by qualified health professionals with systematic training, it is nonetheless a limitation that diagnostic reliability was not investigated. Moreover, although the study aims to investigate the range of PDs, we focused primarily on the most common PDs, which led to less emphasis on how alexithymia applies to the full range of PDs. It is also a limitation that clinical outcome measures were based on patient self-report. However, supplementary assessment of global status was based on a combination of clinician ratings and patient self-reports where overall trends by self-report versus therapist interview were comparable. We also included supplementary self-reports of emotional clarity, and overall trends were comparable. Incomplete data sets represented a limitation to drawing conclusions. Missing data are to be anticipated in naturalistic treatment settings with repeated assessments over long time periods, and paper-based administration of questionnaires and registration of data are vulnerable. The chosen statistical method was appropriate for samples with unbalanced data and long study periods and enabled the utilization of available longitudinal data (59). In order not to overestimate trends, we reported LMM analyses of the total sample and the longitudinal study sample with more frequent measurement occasions and included consideration of possible missing data patterns (60). Being based on available data from a quality registry, our study lacked post-treatment evaluations. As a possible consequence, longitudinal trends may have been underestimated. Because the treatment setting included a relatively diverse range of treatments, it was unclear which aspects of treatment may have contributed to improvements in alexithymia. Finally, the paper has a limitation in that we could not conclude that the change in alexithymia resulted from the treatment, as we did not have a control group or an untreated comparison group.

## Conclusion

Alexithymia is a common emotional deficit among patients with PDs. High levels of alexithymia are associated with a range of mental health difficulties such as anxiety, depression, and increased PD severity, along with reduced quality of life and impaired psychosocial functioning. The present findings illuminate the significant emotional burdens faced by alexithymic individuals. Importantly, alexithymia appears to improve over time during treatment, suggesting that psychological treatments may help improve alexithymia and its associated difficulties. The findings have clinical implications for mental health professionals working with PD patients in tailoring and planning treatment. Future research should aim to evaluate the effectiveness of different treatments and interventions in reducing alexithymia among PD patients.

## Data Availability

The datasets presented in this article are not readily available because the data used in this study are based on a quality register of the Norwegian Network for Personality Disorders. Due to restrictions regarding patient confidentiality, data are only available on specific request. Requests to access these datasets should be directed to the Privacy and Data protection Officer at Oslo University Hospital; personvern@ous-hf.no or the corresponding author, EHK e.h.kvarstein@medisin.uio.no.

## Footnotes

1 Pedersen G, Hummelen B, Gulbrandsen S, Leitemo K, Oftedal E, Bakali JV, et al. Psychometric evaluation of the Norwegian Difficulties in Emotion Regulation Scale in a multi-site sample of patients with personality disorders and personality problems. (2024).

## References

[1] Nemiah JC Sifneos PE. Psychosomatic illness: A problem in communication. Psychother Psychosom. (1970) 18:154–60. doi: 10.1159/000286074 5520658

[2] Taylor GJ Bagby RM. Examining proposed changes to the conceptualization of the alexithymia construct: the way forward tilts to the past. Psychother Psychosom. (2020) 90:145–55. doi: 10.1159/000511988 33285546

[3] Bagby RM Parker JD Taylor GJ. The twenty-item Toronto Alexithymia Scale–I. Item selection and cross-validation of the factor structure. J Psychosom Res. (1994) 38:23–32. doi: 10.1016/0022-3999(94)90005-1 8126686

[4] Fonagy P Luyten P. A developmental, mentalization-based approach to the understanding and treatment of borderline personality disorder. Dev Psychopathol. (2009) 21:1355–81. doi: 10.1017/S0954579409990198 19825272

[5] Gratz KL Roemer L. Multidimensional assessment of emotion regulation and dysregulation: development, factor structure, and initial validation of the difficulties in emotion regulation scale. J Psychopathol Behav Assess. (2004) 26:41–54. doi: 10.1023/B:JOBA.0000007455.08539.94

[6] Sharp C Wall K. DSM-5 level of personality functioning: refocusing personality disorder on what it means to be human. Annu Rev Clin Psychol. (2021) 17:313–37. doi: 10.1146/annurev-clinpsy-081219-105402 33306924

[7] Solbakken OA Hansen RS Havik OE Monsen JT. Assessment of affect integration: Validation of the affect consciousness construct. J Pers Assess. (2011) 93:257–65. doi: 10.1080/00223891.2011.558874 21516584

[8] Winsper C Bilgin A Thompson A Marwaha S Chanen AM Singh SP. The prevalence of personality disorders in the community: a global systematic review and meta-analysis. Br J Psychiatry. (2020) 216:69–78. doi: 10.1192/bjp.2019.166 31298170

[9] Beckwith H Moran PF Reilly J. Personality disorder prevalence in psychiatric outpatients: A systematic literature review. Pers Ment Health. (2014) 8:91–101. doi: 10.1002/pmh.1252 24431304

[10] Kvarstein EH Frøyhaug M Pettersen MS Carlsen S Ekberg A Fjermestad-Noll J. Improvement of personality functioning among people treated within personality disorder mental health services. A longitudinal, observational study [Original Research. Front Psychiatry. (2023) 14:1163347. doi: 10.3389/fpsyt.2023.1163347 37229394 PMC10203961

[11] Hogeveen J Grafman J. Alexithymia. In: Heilman KM Nadeau SE, editors. Handbook of Clinical Neurology, vol. 183. Amsterdam: Elsevier (2021). p. 47–62. doi: 10.1016/B978-0-12-822290-4.00004-9 PMC8456171 34389125

[12] Honkalampi K Hintikka J Tanskanen A Lehtonen J Viinamäki H. Depression is strongly associated with alexithymia in the general population. J Psychosom Res. (2000) 48:99–104. doi: 10.1016/S0022-3999(99)00083-5 10750635

[13] Leweke F Leichsenring F Kruse J Hermes S. Is alexithymia associated with specific mental disorders. Psychopathology. (2011) 45:22–8. doi: 10.1159/000325170 22123513

[14] Bach M de Zwaan M Ackard D Nutzinger DO Mitchell JE. Alexithymia: relationship to personality disorders. Compr Psychiatry. (1994) 35:239–43. doi: 10.1016/0010-440x(94)90197-x 8045115

[15] De Panfilis C Ossola P Tonna M Catania L Marchesi C. Finding words for feelings: The relationship between personality disorders and alexithymia. Pers Individ Dif. (2015) 74:285–91. doi: 10.1016/j.paid.2014.10.050

[16] Nicolò G Semerari A Lysaker PH Dimaggio G Conti L D’Angerio S. Alexithymia in personality disorders: correlations with symptoms and interpersonal functioning. Psychiatry Res. (2011) 190:37–42. doi: 10.1016/j.psychres.2010.07.046 20800288

[17] Joyce AS Fujiwara E Cristall M Ruddy C Ogrodniczuk JS. Clinical correlates of alexithymia among patients with personality disorder. Psychother Res. (2013) 23:690–704. doi: 10.1080/10503307.2013.803628 23731378

[18] Preece DA Mehta A Petrova K Sikka P Bjureberg J Becerra R. Alexithymia and emotion regulation. J Affect Disord. (2023) 324:232–8. doi: 10.1016/j.jad.2022.12.065 36566943

[19] Simonsen S Eikenæs IU-M Bach B Kvarstein EH Gondan M Møller SB. Level of alexithymia as a measure of personality dysfunction in avoidant personality disorder. Nordic J Psychiatry. (2021) 75:266–74. doi: 10.1080/08039488.2020.1841290 33146059

[20] Lysaker PH Olesek K Buck K Leonhardt BL Vohs J Ringer J. Metacognitive mastery moderates the relationship of alexithymia with cluster C personality disorder traits in adults with substance use disorders. Addictive Behav. (2014) 39:558–61. doi: 10.1016/j.addbeh.2013.11.007 24300836

[21] Sexton MC Sunday SR Hurt S Halmi KA. The relationship between alexithymia, depression, and axis II psychopathology in eating disorder inpatients. Int J Eat Disord. (1998) 23:277–86. doi: 10.1002/(SICI)1098-108X(199804)23:3<277::AID-EAT5>3.0.CO;2-G 9547662

[22] Honkalampi K Hintikka J Antikainen R Lehtonen J Viinamäki H. Alexithymia in patients with major depressive disorder and comorbid cluster C personality disorders: A 6-Month follow-up study. J Pers Disord. (2001) 15:245–54. doi: 10.1521/pedi.15.3.245.19211 11406996

[23] New AS Rot M Ripoll LH Perez-Rodriguez MM Lazarus S Zipursky E. Empathy and alexithymia in borderline personality disorder: clinical and laboratory measures. J Pers Disord. (2012) 26:660–75. doi: 10.1521/pedi.2012.26.5.660 23013336

[24] Zlotnick C Mattia JI Zimmerman M. The relationship between posttraumatic stress disorder, childhood trauma and alexithymia in an outpatient sample. J Trauma Stress. (2001) 14:177–88. doi: 10.1023/A:1007899918410

[25] De Rick A Vanheule S Verhaeghe P. Alcohol addiction and the attachment system: an empirical study of attachment style, alexithymia, and psychiatric disorders in alcoholic inpatients. Subst Use Misuse. (2009) 44:99–114. doi: 10.1080/10826080802525744 19137485

[26] Cameron K Ogrodniczuk J Hadjipavlou G. Changes in alexithymia following psychological intervention: a review. Harvard Rev Psychiatry. (2014) 22:162–78. doi: 10.1097/hrp.0000000000000036 24736520

[27] Ogrodniczuk JS Joyce AS Piper WE. Change in alexithymia in two dynamically informed individual psychotherapies. Psychother Psychosom. (2013) 82:61–3. doi: 10.1159/000341180 23147381

[28] Ogrodniczuk JS Piper WE Joyce AS. Effect of alexithymia on the process and outcome of psychotherapy: a programmatic review. Psychiatry Res. (2011) 190:43–8. doi: 10.1016/j.psychres.2010.04.026 20471096

[29] Löf J Clinton D Kaldo V Rydén G. Symptom, alexithymia and self-image outcomes of Mentalisation-based treatment for borderline personality disorder: a naturalistic study. BMC Psychiatry. (2018) 18:185. doi: 10.1186/s12888-018-1699-6 29890960 PMC5996479

[30] Rossi R Corbo D Magni LR Pievani M Nicolò G Semerari A. Metacognitive interpersonal therapy in borderline personality disorder: Clinical and neuroimaging outcomes from the CLIMAMITHE study-A randomized clinical trial. Pers Disorders: Theory Res Treat. (2023) 14:452–66. doi: 10.1037/per0000621 37227866

[31] Bianchini V Cofini V Curto M Lagrotteria B Manzi A Navari S. Dialectical behaviour therapy (DBT) for forensic psychiatric patients: An Italian pilot study. Crim Behav Ment Health. (2019) 29:122–30. doi: 10.1002/cbm.2102 30648303

[32] Simonsen S Popolo R Juul S Frandsen FW Sørensen P Dimaggio G. Treating avoidant personality disorder with combined individual metacognitive interpersonal therapy and group mentalization-based treatment: A pilot study. J Nervous Ment Dis. (2022) 210(3):163–71. doi: 10.1097/NMD.0000000000001432 34710894

[33] Wilberg T Pedersen G Bremer K Johansen MS Kvarstein EH. Combined group and individual therapy for patients with avoidant personality disorder—A pilot study [Original Research. Front Psychiatry. (2023) 14:1181686. doi: 10.3389/fpsyt.2023.1181686 37215654 PMC10192633

[34] Massaal-van-der-Ree LY Eikelenboom M Hoogendoorn AW Thomaes K van Marle HJF. Cluster B versus cluster C personality disorders: A comparison of comorbidity, suicidality, traumatization and global functioning. Behav Sci. (2022) 12. doi: 10.3390/bs12040105 PMC9031793 35447677

[35] Shah R Zanarini MC. Comorbidity of borderline personality disorder: current status and future directions. Psychiatr Clinics North America. (2018) 41:583–93. doi: 10.1016/j.psc.2018.07.009 30447726

[36] Pedersen G Wilberg T Hummelen B Kvarstein EH. The Norwegian network for personality disorders – development, contributions and challenges through 30 years. Nordic J Psychiatry. (2022) 77(5):512–20. doi: 10.1080/08039488.2022.2147995 36409693

[37] Øvstebø RB Pedersen G Wilberg T Røssberg JI Dahl HSJ Kvarstein EH. Countertransference in the treatment of patients with personality disorders: A longitudinal study. Psychother Res. (2023), 1–15. doi: 10.1080/10503307.2023.2279645 37963354

[38] Sheehan DV Lecrubier Y Janavs J Knapp E Weiller E Bonora LI. Mini International Neuropsychiatric Interview (MINI). Tampa, Florida and Paris, France: University of South Florida Institutt for Research in Psychiatry and INSERM-Hôpital de la Salpétrière (1994).

[39] First MB Williams JBW Karg RS Spitzer RL. Structured clinical interview for DSM-5 clinical version (SCID-5-PD). Arlington, VA: American Psychiatric Association (2016).

[40] Pedersen G Karterud S Hummelen B Wilberg T. The impact of extended longitudinal observation on the assessment of personality disorders. Pers Ment Health. (2013) 7:277–87. doi: 10.1002/pmh.1234 24343977

[41] Spitzer RL. Psychiatric diagnosis: are clinicians still necessary? Comprehensive Psychiatry. (1983) 24:399–411. doi: 10.1016/0010-440X(83)90032-9 6354575

[42] Spitzer RL Kroenke K Williams JB Lowe B. A brief measure for assessing generalized anxiety disorder: the GAD-7. Arch Intern Med. (2006) 166:1092–7. doi: 10.1001/archinte.166.10.1092 16717171

[43] Kroenke K Spitzer RL Williams JB Monahan PO Lowe B. Anxiety disorders in primary care: prevalence, impairment, comorbidity, and detection. Ann Intern Med. (2007) 146:317–25. doi: 10.7326/0003-4819-146-5-200703060-00004 17339617

[44] Kroenke K Spitzer RL Williams JB. The PHQ-9: validity of a brief depression severity measure. J Gen Intern Med. (2001) 16:606–13. doi: 10.1046/j.1525-1497.2001.016009606.x PMC1495268 11556941

[45] Lee PW Schulberg HC Raue PJ Kroenke K. Concordance between the PHQ-9 and the HSCL-20 in depressed primary care patients. J Affect Disord. (2007) 99:139–45. doi: 10.1016/j.jad.2006.09.002 17049999

[46] Ettman CK Abdalla SM Cohen GH Sampson L Vivier PM Galea S. Prevalence of depression symptoms in US adults before and during the COVID-19 pandemic. JAMA Netw Open. (2020) 3:e2019686. doi: 10.1001/jamanetworkopen.2020.19686 32876685 PMC7489837

[47] Lakhan R Agrawal A Sharma M. Prevalence of depression, anxiety, and stress during COVID-19 pandemic. J Neurosci Rural Pract. (2020) 11:519–25. doi: 10.1055/s-0040-1716442 PMC7595780 33144785

[48] Pedersen G Normann-Eide E Eikenæs IU-M Kvarstein EH Wilberg T. Psychometric evaluation of the Norwegian Toronto Alexithymia Scale (TAS-20) in a multisite clinical sample of patients with personality disorders and personality problems. J Clin Psychol. (2022) 78:1118–36. doi: 10.1002/jclp.23270 34716595

[49] Hallion LS Steinman SA Tolin DF Diefenbach GJ. Psychometric properties of the difficulties in emotion regulation scale (DERS) and its short forms in adults with emotional disorders. Front Psychol. (2018) 9:539. doi: 10.3389/fpsyg.2018.00539 29725312 PMC5917244

[50] Ritschel LA Tone EB Schoemann AM Lim NE. Psychometric properties of the Difficulties in Emotion Regulation Scale across demographic groups. psychol Assess. (2015) 27:944–54. doi: 10.1037/pas0000099 25774638

[51] van Asselt AD Dirksen CD Arntz A Giesen-Bloo JH Severens JL. The EQ-5D: A useful quality of life measure in borderline personality disorder? Eur Psychiatry. (2009) 24:79–85. doi: 10.1016/j.eurpsy.2008.11.001 19095421

[52] Saarni SI Harkanen T Sintonen H Suvisaari J Koskinen S Aromaa A. The impact of 29 chronic conditions on health-related quality of life: a general population survey in Finland using 15D and EQ-5D. Qual Life Res. (2006) 15:1403–14. doi: 10.1007/s11136-006-0020-1 16960751

[53] American Psychiatric Association. Diagnostic and statistical manual of mental disorders: DSM-IV. Washington D.C.: American Psychiatric Association (1994).

[54] Pedersen G Urnes Ø Hummelen B Wilberg T Kvarstein EH. Revised manual for the Global Assessment of Functioning scale. Eur Psychiatry. (2018) 51:16–9. doi: 10.1016/j.eurpsy.2017.12.028 29510296

[55] Pedersen G Hagtvet KA Karterud S. Generalizability studies of the Global Assessment of Functioning–Split version. Compr Psychiatry. (2007) 48:88–94. doi: 10.1016/j.comppsych.2006.03.008 17145287

[56] Pedersen G Karterud S. The symptom and function dimensions of the Global Assessment of Functioning (GAF) scale. Compr Psychiatry. (2012) 53:292–8. doi: 10.1016/j.comppsych.2011.04.007 21632038

[57] IBMCorp. IBM SPSS Statistics for Windows. Armonk, NY: IBM Corp (2017).

[58] Heck RH Scott TL Lynn TN. Multilevel and longitudinal modeling with IBM SPSS. New York: Routledge (2010).

[59] Singer JD Willett JB. Applied longitudinal data analysis. New York: Oxford University Press (2003).

[60] Hedeker D Gibbons RD. Application of random-effects pattern-mixture models for missing data in longitudinal studies. psychol Methods. (1997) 2:64–78. doi: 10.1037/1082-989X.2.1.64

[61] Cohen J. Statistical power analysis for the behavioral sciences. 2 ed. New York: Routledge (1988). doi: 10.4324/9780203771587

[62] Bo S Sharp C Fonagy P Kongerslev M. Hypermentalizing, attachment, and epistemic trust in adolescent BPD: Clinical illustrations. Pers Disorders: Theory Res Treat. (2017) 8:172–82. doi: 10.1037/per0000161 26691672

[63] Herpertz SC Bertsch K. The social-cognitive basis of personality disorders. Curr Opin Psychiatry. (2014) 27:73–7. doi: 10.1097/yco.0000000000000026 24270477

[64] Semerari A Colle L Pellecchia G Buccione I Carcione A Dimaggio G. Metacognitive dysfunctions in personality disorders: correlations with disorder severity and personality styles. J Pers Disord. (2014) 28:751–66. doi: 10.1521/pedi_2014_28_137 24689762

[65] Moroni F Procacci M Pellecchia G Semerari A Nicolò G Carcione A. Mindreading dysfunction in avoidant personality disorder compared with other personality disorders. J Nervous Ment Dis. (2016) 204:752–7. doi: 10.1097/nmd.0000000000000536 27227557

[66] Millon T. Disorders of Personality: DSM-III: Axis II. New York: Wiley (1981).

[67] Preece D Becerra R Robinson K Dandy J. Assessing alexithymia: psychometric properties and factorial invariance of the 20-item Toronto alexithymia scale in nonclinical and psychiatric samples. J Psychopathol Behav Assess. (2018) 40:276–87. doi: 10.1007/s10862-017-9634-6

[68] Li S Zhang B Guo Y Zhang J. The association between alexithymia as assessed by the 20-item Toronto Alexithymia Scale and depression: A meta-analysis. Psychiatry Res. (2015) 227:1–9. doi: 10.1016/j.psychres.2015.02.006 25769520

[69] Marchesi C Brusamonti E Maggini C. Are alexithymia, depression, and anxiety distinct constructs in affective disorders? J Psychosom Res. (2000) 49:43–9. doi: 10.1016/S0022-3999(00)00084-2 11053603

[70] McMain S Links PS Guimond T Wnuk S Eynan R Bergmans Y. An exploratory study of the relationship between changes in emotion and cognitive processes and treatment outcome in borderline personality disorder. Psychother Res. (2013) 23:658–73. doi: 10.1080/10503307.2013.838653 24156526

[71] Soeteman DI Hakkaart-van-Roijen L Verheul R Busschbach JJ. The economic burden of personality disorders in mental health care. J Clin Psychiatry. (2008) 69:259–65. doi: 10.4088/jcp.v69n0212 18363454

[72] Sveen CA Pedersen G Ulvestad DA Zahl KE Wilberg T Kvarstein EH. Societal costs of personality disorders among treatment-seeking patients in Norway: the relative contribution of specific DSM-5 categories. Eur Arch Psychiatry Clin Neurosci. (2023) 274:139–49. doi: 10.1007/s00406-023-01655-1 PMC10786999 37598131

